# Imaging of the Eustachian tube and its function: a systematic review

**DOI:** 10.1007/s00234-016-1663-4

**Published:** 2016-02-27

**Authors:** M. E. Smith, D. J. Scoffings, J. R. Tysome

**Affiliations:** Department of ENT Surgery, Addenbrooke’s Hospital, Cambridge University Hospitals NHS Foundation Trust, Hills Road, Cambridge, CB2 0QQ UK; Department of Radiology, Addenbrooke’s Hospital, Cambridge University Hospitals NHS Foundation Trust, Hills Road, Cambridge, CB2 0QQ UK

**Keywords:** Eustachian tube, Radiology, Diagnostic imaging, Anatomy

## Abstract

**Introduction:**

The Eustachian tube is a complex and inaccessible structure, which maintains middle ear ventilation to facilitate transmission of sound from the tympanic membrane to the cochlea. A renewed interest in treatments for eustachian tube dysfunction has led to a demand for methods of imaging the Eustachian tube, and assessing tube opening non-invasively. This review aims to summarise the use of imaging in the anatomical assessment of the Eustachian tube, and to explore how radiological techniques can be used to assess tube function.

**Methods:**

A systematic review of the literature was performed with narrative data analysis.

**Results:**

With high-resolution images, the soft and bony anatomy of the Eustachian tube can be assessed in detail. CT and MRI are best suited to identifying features associated with obstructive or patulous Eustachian tube dysfunction, though true assessments of function have only been achieved with contrast enhanced radiographs and scintigraphy. A single modality has yet to provide a complete assessment. No test has entered routine clinical use, but further development and research is underway.

**Conclusion:**

Significant information can be gained from imaging the Eustachian tube, and as faster acquisition techniques are developed, it is possible that dynamic imaging of tubal opening could play an important role in the assessment of patients with ET dysfunction.

## Background

A normally functioning Eustachian tube (ET) is essential for maintaining a healthy middle ear and normal hearing. Failure of the ET to function correctly is a common cause of morbidity in both adults and children, and yet the anatomy, function and imaging options relating to the tube are still areas poorly understood by otolaryngologists and radiologists alike. Due to recent developments in the surgical management of Eustachian tube disorders, in particular with the introduction of balloon dilation of the ET, it is timely to review this important structure again, and assess what imaging can add in terms of both our understanding and diagnosis of Eustachian tube pathology.

The ET is a narrow, epithelial-lined tube extending from the middle ear to the nasopharynx. It is passively closed, but normally opens under the control of paratubal muscles during actions such as swallowing [1]. The ET fulfils three main roles, which together facilitate middle ear health and the transmission of sound from the tympanic membrane to the cochlea. The first function is equalisation of middle ear pressure to the ambient atmospheric pressure: a necessity due to external pressure fluctuations and mucosal gas exchange. The second function is to permit the mucociliary clearance of middle ear secretions and the third is to prevent the retrograde travel of speech sounds and pathogen-laden secretions up the ET from the nasopharynx [2].

Failure of the above-mentioned actions is considered ET ‘dysfunction’ (ETD), which is usually classified as either obstructive or patulous. Obstructive ETD results from inadequate tube opening, caused by either dynamic dysfunction (muscular failure) or, more commonly, from obstruction of the tubal lumen by intrinsic changes or associated structures such as hypertrophic adenoids. Failure of the tube to open adequately typically results in the patient complaining of ear fullness, ‘popping’ sounds, discomfort, muffled hearing and tinnitus [3]. Tympanic membrane retraction, cholesteatoma, perforation and middle ear effusion are all associated with ETD [2]. Patulous ETD is far less common, and results from the ET remains permanently open. Patients with patulous ETD often complain of a feeling of pressure in their ear or of abnormal hearing of their own voice (autophony) [4].

There are two clinical requirements for radiological assessment of the ET: a reliable test of ET function and detailed anatomical imaging of the ET and its surrounding structures for surgical planning. The inaccessibility of the ET has contributed to the fact that there is currently no reliable test for determining ET function [5], meaning that many diagnoses are based on clinical history alone. The lack of reliable outcome measures for ETD has hindered research into new treatments [5, 6] and complicated patient selection for new therapies. Imaging may provide a useful non-invasive solution to assessing ET function.

Surgeons increasingly require imaging in pre-operative planning for complex skull base surgery, for ET resection in malignancy and for new interventions for ETD such as balloon dilatation tuboplasty [7]. The proximity of the ET to the internal carotid artery and other skull base structures, combined with inter-patient anatomical variation, can make surgery in this area hazardous.

In addition to the evolving clinical need, imaging has been instrumental in developing our understanding of the ET’s structure and dynamic function, providing valuable information where cadaveric dissection and in vivo endoscopy have failed. It is likely that new imaging techniques will continue to advance our understanding of ETD.

This systematic review aims to answer the following questions:

1. Which anatomical structures of the ET can be defined radiologically?
2. What are the radiological features of obstructive ETD?
3. What are the radiological features of patulous ETD?
4. How can imaging be used as a measure of ET function (a) clinically and (b) in the research setting?

The ET also plays an important role in the spread of malignancies such as nasopharyngeal carcinoma, with imaging essential for staging and management planning [8, 9]. However, this has been excluded from this review, which focuses on ETD. Detailed descriptions of scanning techniques have also been omitted, but are available in the referenced articles.

## Methods

### Identification of studies

A comprehensive search strategy was developed to capture all published material relating to imaging of the Eustachian tube (available in supplementary material). MEDLINE, EMBASE and CENTRAL were searched on 11 February 2015. The reference lists of relevant studies were searched, and in addition, citation searches were performed.

### Study selection

To be considered a viable clinical technique, authors must, as a minimum, have described test results in both ETD cases and healthy controls. Anatomical descriptions, techniques in development and non-controlled series were considered separately. Only publications from 1970 onwards were included as being relevant to contemporary practice. Isolated case reports, animal studies and descriptions relating to cancer were excluded.

### Data extraction

Publications were assessed in the context of the questions stated earlier. Where possible, quantitative data were extracted for comparison.

### Quality assessment

Sample size, risk of bias, generalisability and clinical relevance were assessed.

### Data synthesis

The aims of this review dictated a qualitative data analysis through a narrative approach.

## Results

**Fig. 1:**
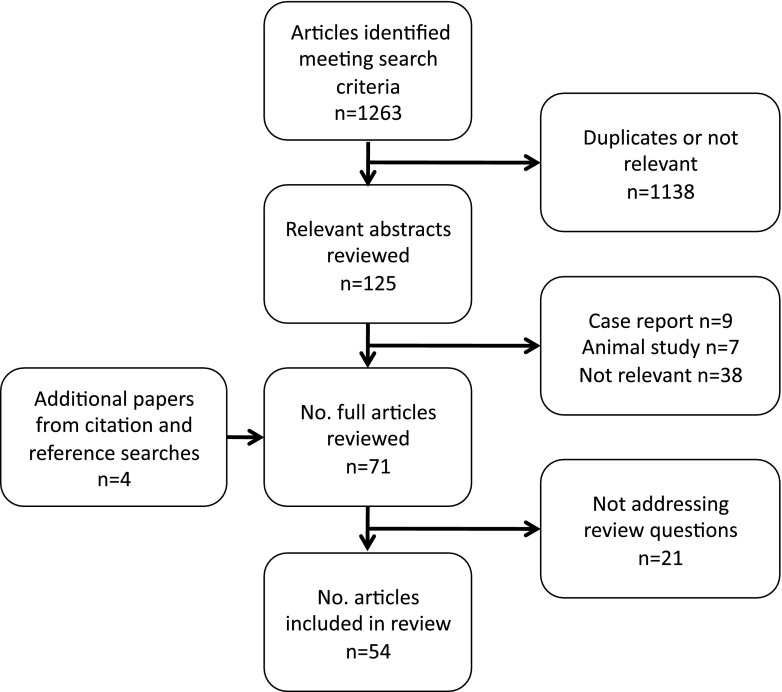
Literature search flow chart

Due to study heterogeneity, formal data synthesis for any of the questions was not possible and a narrative review was performed for each. The search results are summarised in Fig. 1.

### Which anatomical structures of the Eustachian tube can be defined radiologically?

The ET can be considered a continuous organ with the middle ear and mastoid cells. The tube is usually divided into three sections when described anatomically: cartilaginous, osseous and junctional, where the two main parts overlap. Particularly in adults, the ET can assume a gently curving, inverted ‘S’ shape, although there is a range from almost straight to acutely curved [10]. Combined with its angle, this shape means the ET is not easily assessed in axial or coronal planes and can be difficult to visualise in its entirety in any single plane, even using multiplanar reconstruction to obtain a plane perpendicular to the long axis of the ET [11, 12]. A number of identified papers address the imaging of the anatomical structures of the ET, almost all using CT and MRI. Whilst some present comparative findings from different dissected cadaver specimens, none comprehensively compare modalities or histological sections to imaging within the same specimen.

#### Cartilaginous ET

The cartilaginous tube is closely associated with the skull base, and runs in a depression, the ‘sulcus tubae’, between the greater wing of the sphenoid and the petrous part of the temporal bone [2, 11]. The antero-medial end protrudes into the nasopharynx, forming the torus tubarius around the tubal ostium. The torus tubarius may contain accessory cartilage and is covered with a thick layer of epithelium. It is attached to a protuberance of the medial pterygoid lamina [2, 11] and forms a landmark visible on CT and MR where it protrudes into the aerated nasopharynx.

#### Length and angle

There is considerable growth of the ET in the first few years of life, and it continues to develop and enlarge into early adulthood. Takasaki et al. found a linear relationship between ET cartilage volume and age, with an increase in volume of 20 mm^3^ per year after birth, up to the age of 20 [13]. Based on in vivo measurements from CT scans, the total length of the cartilaginous and bony ET is approximately 38 mm in children (mean age of 5 years), compared to 43 mm in adults [14]. These values are slightly higher than those seen in cadaver studies, though the difference with age is consistent [15].

**Fig. 2:**
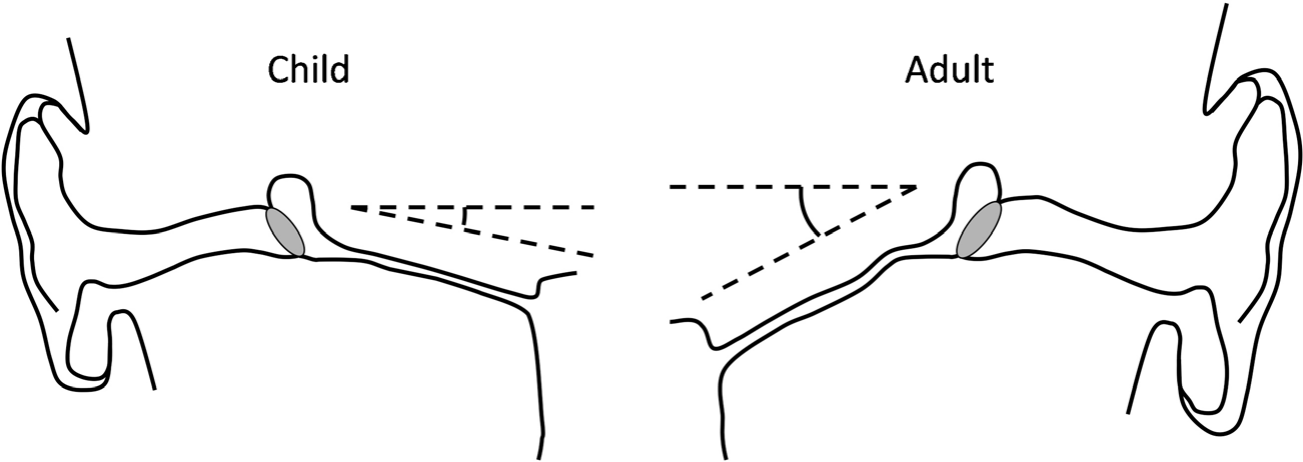
Schematic diagram showing the relative angles of the ET from the horizontal plane in children and adults

In adults, the ET forms an average angle of 34–36 ° with the horizontal plane, using the bony palate as a reference, although there is variation between individuals [16, 17]. The ET axis with reference to the sagittal plane is approximately 42 ° [17]. Takasaki et al. measured the ET angle against Reid’s standard plane (a plane passing through the inferior orbital margin and the upper margin of the external auditory meatus) using CT, and found that in young children, the ET angle is closer to horizontal than in adults, but that there was no significant difference between the angle in children with otitis media with effusion (OME) and controls [14]. The shallower angle of the ET in young children (as little as 10 ° in some reports) has been widely confirmed, and is hypothesised as being a predisposing factor for middle ear inflammation and infections in this group, due to an increased risk of reflux of nasopharyngeal secretions and pathogens [2]. Figure 2 demonstrates the difference in angle of the paediatric and adult ET.

#### Lumen and isthmus

The healthy ET lumen is collapsed at rest, and consequently cannot be imaged in this state. Many have likened the ET lumen to two cones, meeting at their tips to form a narrowing of around 1.5 mm diameter: the isthmus [17]. The location of this isthmus has been debated, but it appears to occur two thirds of the way along the ET from the nasopharyngeal end, in the cartilaginous part, just before the junctional region [2, 10]. Using CT with contrast injected into the ETs of cadavers, Oberascher et al. characterised the lumen shape as oval, except for at the isthmus where it was found to be circular [18]. Using the same technique, they also found that the isthmus was absent in infants [19].

#### Mucosa

The lumen is lined with ciliated pseudostratified columnar epithelium to assist with the clearance of material into the nasopharynx [2]. The mucosal lining contains folds and glands inferiorly, and can become thickened, contributing to obstructive ETD. Measuring mucosal thickness radiologically is problematic, and Naito et al. found that T2-weighted MRI demonstrated the normal variable thickness well, but tended to over-estimate thickness when compared to histological sectioning [20]. Helweg et al. used endoluminal ultrasound and found good resolution of the mucosa, though the technique was not developed further [21]. The mucosa cannot be clearly defined on CT [17].

#### Muscles

**Fig. 3:**
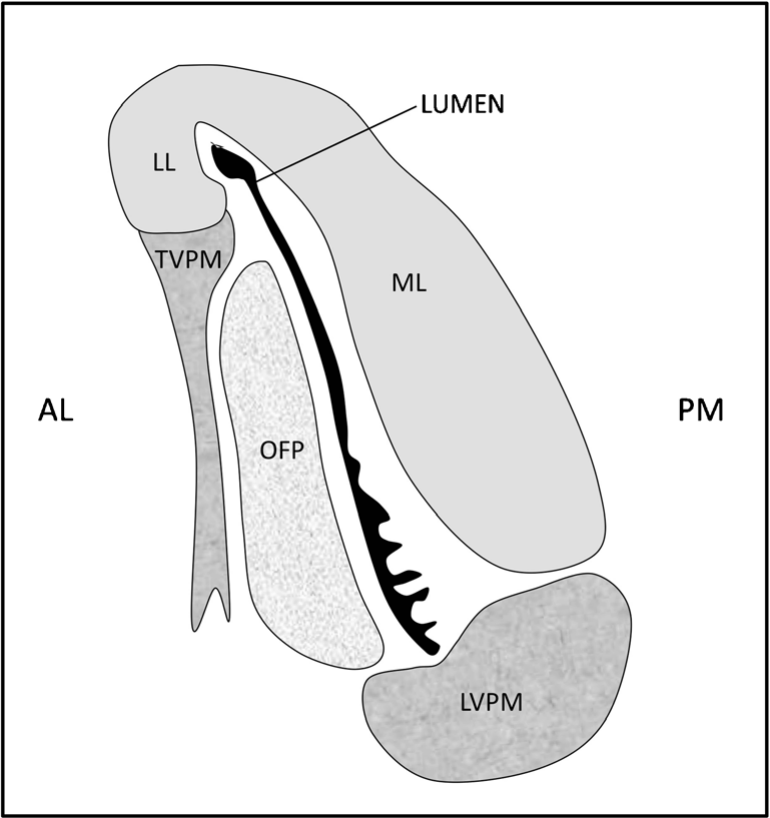
A schematic of a transverse section through the ET in the mid-cartilaginous section, as seen in the closed state, with the collapsed lumen shown in *black*. The mucosal folds in the inferior part of the lumen can be seen. *ML* medial lamina of the cartilage, *LL* lateral lamina, *OFP* Ostmann fat pad, *TVPM* tensor veli palatini muscle, *LVPM* levator veli palatini muscle. Orientation: *AL* antero-lateral, *PM* postero-medial

Four muscles are associated with the ET: the tensor veli palatini, the levator veli palatini, the tensor tympani and the salpingopharyngeus. The ET is passively closed at rest, and it is thought that the tensor veli palatini plays the main role in opening the lumen [1]. This muscle arises in two parts from the greater wing of the sphenoid bone and from the fibrocartilaginous ET, before passing downwards to hook around the pterygoid hamulus and insert into the soft palate aponeurosis. The levator veli palatini arises from the inferior aspect of the petrous temporal bone, running below and parallel to the ET floor. It is thought only to be related to the ET by loose connective tissue, opening the ET during contraction by rotating the medial part of the cartilage with its increased bulk [10]. The relationship between the ET and these muscles is shown in Fig. 3.

The salpingopharyngeus arises from the medial and inferior part of the cartilaginous ET. This small muscle, which is often poorly formed, courses inferiorly embedded within connective tissue, to insert into the pharyngeal wall [22, 23]. The tensor tympani arises from the cartilaginous ET and sphenoid bone, and receives fibres from the tensor veli palatini, before ending in a tendon that inserts into the manubrium of the malleus. Despite their proximity, neither the salpingopharyngeus or tensor tympani are thought to influence ET opening [2, 23].

**Fig. 4:**
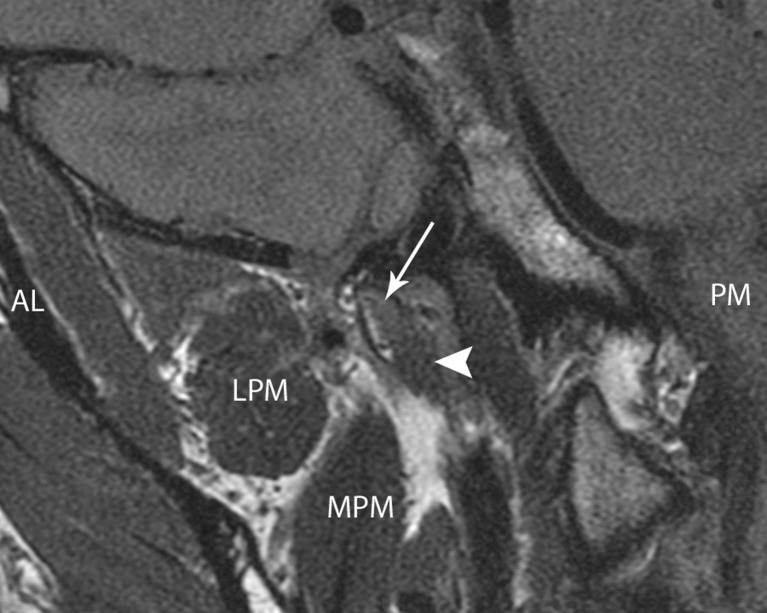
Coronal oblique proton density MRI of the right ET and paratubal structures in a 36-year-old healthy male volunteer. The tubal cartilage is indicated by the *arrow*, the levator palatini muscle by the *arrowhead. LPM* lateral pterygoid muscle. *MPM* medial pterygoid muscle. *AL* and *PM* denote antero-lateral and posteromedial orientation of the imaging plane

The tensor and the levator veli palatini muscles are usually well visualised using MRI [17, 24, 25], where they can be seen to be separated by a layer of fat [26] and their dimensions can be assessed [27] (Fig. 4). Electromyographic and other studies have been inconclusive as to the role of the paratubal muscles in obstructive ETD [28, 29]. In humans, cranial nerve dysfunction has not been linked to ETD, though abnormal muscle function is thought to be the reason that ETD is prevalent in those with a cleft palate.

#### Cartilage

The fibro-cartilage of the ET extends from the nasopharyngeal opening to firmly attach to the osseous orifice with fibrous bands. The cartilage forms the roof and medial wall of the ET, and is shaped like an ‘inverted J’, with the superior hook section rich in elastin and acting as a hinge during ET opening [30]. T1- and T2-weighted MRI in the oblique parasagittal plane is consistently superior to other modalities for imaging the ET cartilage [16], with short TI inversion recovery (STIR) images being of the highest quality [25]. Visualisation of the ET cartilage with MRI is poor in some individuals, particularly with advanced age [25], and on CT it often cannot be identified, appearing isodense with surrounding soft tissues [16].

#### Ostmann fat pad

**Fig. 5:**
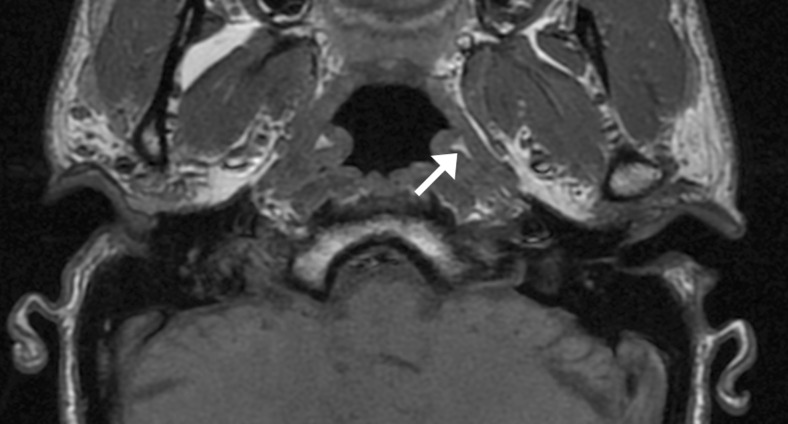
Axial T1 spin echo MRI of the nasopharynx in a 36-year-old healthy male volunteer. Ostmann fatty tissue is indicated on the left by the *arrow*

Ostmann fat pad is an area of fatty tissue running the length of the cartilaginous ET, infero-lateral to the lumen, that is thought to play a role in tube closure [2]. It is poorly visualised on CT [31], but consistently seen with T1-weighted MRI [24] (Fig. 5). Amoodi et al. found Ostmann fat pad to be best visualised on axial T1-weighted post-gadolinium MR images, and calculated its surface area to demonstrate that unlike other soft tissue structures around the ET, it shrinks with adult ageing [27].

#### Junctional ET, Osseous ET

The lateral third of the ET is osseous, lying within the petrous temporal bone. It joins the cartilaginous ET in a junctional region where the two overlap by 3–4 mm. The osseous part of the ET has been studied less than the cartilaginous part. Temporal bone CT scanning is routinely performed to assess middle and inner ear structures; and in many ears, air can be seen in the lumen of the osseous part of the ET [32].

Jen et al. found half of petrous apices to be pneumatised in CT images, and of these, 92 % featured a peritubal cell appearing to directly communicate with the tubal lumen [33]. This correlates well with histological assessments, helping to validate the accuracy of the images [34]. Jadhav et al. also reported the detailed assessment of peritubal cells using cone beam CT [35], finding that the cells may open at any point along the ET, although this most frequently occurs posterolaterally. Peritubal cells linked to the ET lumen are potential points for CSF leakage after ear and skull base surgery such as the translabyrinthine approach for vestibular schwannoma resection.

#### Relationship to the carotid artery

The horizontal segment of the internal carotid artery courses antero-medially along the long axis of the petrous part of temporal bone, and is crossed by the ET as it descends medially from the middle ear [36]. The relationship between the internal carotid artery and the ET has received significant interest in recent years with the development of new treatments for ETD, and in particular, balloon Eustachian tuboplasty (BET). BET is an ET dilatation technique using a high-pressure balloon catheter, similar to those employed in angioplasty. CT provides the best structural resolution [37–39], and CT angiography can provide further detail if required [36]. Some institutions routinely request CT imaging prior to performing BET to look for arterial dehiscence adjacent to the ET, as this may put the artery at risk. However, many surgeons consider this unnecessary, as commercially available ET balloon catheters introduced via the nasopharynx do not reach this far. Abdel Aziz et al. found carotid canal dehiscence in 6.3 % patients undergoing BET, all of whom went on to have the procedure without complication [37]. In contrast, Tisch et al. found no dehiscence in a total of 2000 scans, with a mean thickness of the carotid canal wall of 1.02 ± 0.29 mm [38].

**Table:** 

**Summary**: **Which anatomical structures of the Eustachian tube can be defined radiologically**? • Bone, cartilage and soft tissue elements of the ET can be visualised radiologically using CT and T1- and T2-weighted MRI • No single modality provides detail of all structures • The lumen is collapsed at rest and cannot be assessed in this state • There is no role for plain film radiography or US in the anatomical assessment of the ET	

### What are the radiological features of obstructive ETD?

The imaging findings associated with ETD have not been extensively described, with only six published papers identified as describing features detectable on imaging that are suggestive of ETD [12, 24, 32, 40–42]. All except one [24] are based on CT findings (Table 1).

**Table 1:** Features of obstructive ETD on imaging

Paper	Imaging modality	Cohort: patients (ears)	Non-imaging comparator	Key findings
Kanzaki 1985 [32]	CT (Obtained in a semi-axial plane)	40 (40) MEE ± VT 10 (10) COM	None	Immediately after grommet insertion and fluid drainage the bony part of ET appeared open Cartilaginous part of ET was always closed
Conticello 1989 [40]	CT (Axial)	COM (*n* not specified) Controls with inner ear disease	None	Reduced lumen size seen in COM Few details reported
Yoshida 2007 [12]	CT (MPR)	25 (38) Obstructive ETD 20 (40) controls	Manometry (passive opening pressure)	ETD cases had a smaller ET bony framework (particular in mid section) and the mucosal thickness was greater No difference in length Isthmus hard to image
Liang 2009 [41]	CT (MPR)	61 (63) MEE Contralateral ear controls	Tympanogram	Soft tissue seen in tympanic orifice in ears with OME Good correlation of CT findings with tympanography
Tsai 2010 [42]	CT (3D reconstruction)	10 (10) Cholesteatoma Contralateral ear controls	None	3D reconstruction is effective with high level of detail ET angle is significantly smaller in diseased ears, otherwise no differences noted
Lukens 2012 [24]	MRI T2 turbo spin echo and T1 gradient echo sequences	16 (26) symptomatic ETD (6) contralateral ear controls	Tympanogram—not clear data presented	Eustachian tube opening during Valsalva can be visualised Most soft tissue structures easily viewed

*MPR* multiplanar reconstruction, *COM* chronic otitis media (infection or inflammation of the middle ear), *VT* ventilation tube (grommet), *MEE* middle ear effusion *OME* otitis media with effusion

Two groups found that the bony channel for the Eustachian tube within the temporal bone was of reduced cross sectional size in individuals with obstructive ETD [12, 40], and one group found that the tube ran at a shallower angle in diseased ears [42]. Kanzaki et al. highlight one of the weaknesses of using CT to assess soft tissues; as by scanning pre- and post-grommet insertion, they demonstrated that mucus and fluid cannot easily be distinguished from soft tissue obstructing the lumen [32]. After grommet insertion, they could localise what appeared to be mucosal thickening in some patients with ETD, a finding shared with Liang et al. who found that these mucosal changes correlated well with tympanometry results (tympanometry measures middle ear pressure, which is close to atmospheric pressure if the ET is functioning normally) [41].

**Fig. 6:**
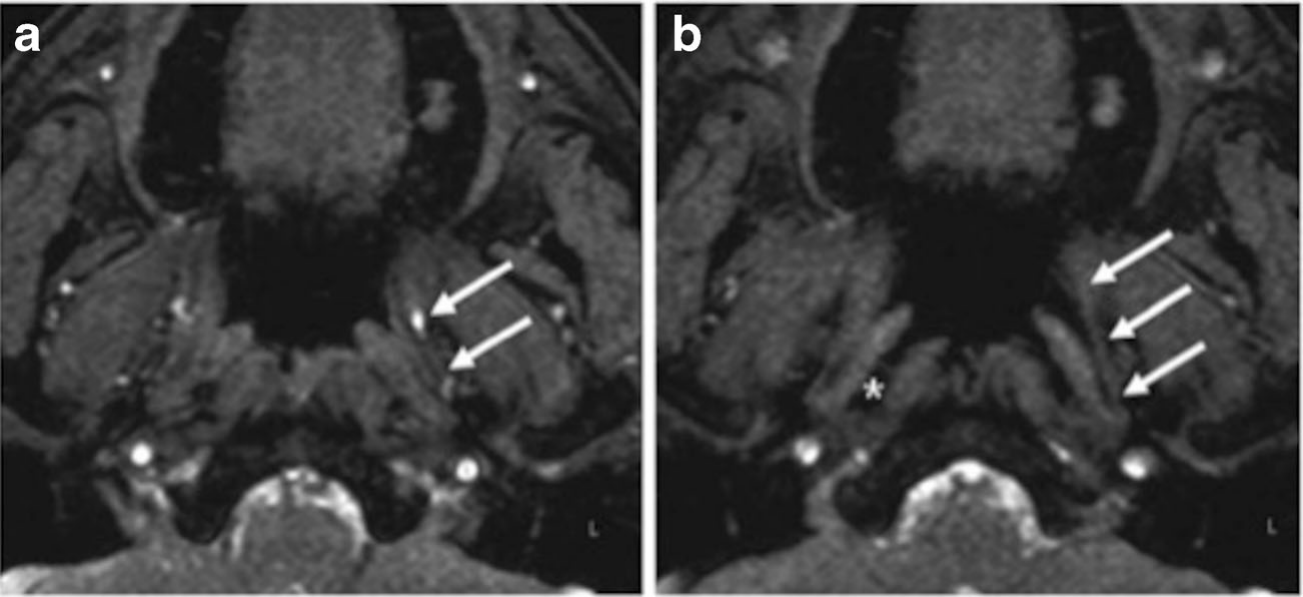
**a**, **b** TFE-SPIR (spectral presaturation with inversion recovery) sequence in neutral position (*left*) and during a Valsalva manoeuvre (*right*). Complete opening of the left ET (*arrows*) and distension of the pharyngeal recess of Rosenmüller (*asterisk*) are visible. Taken from Lukens et al. 2012 [24], with permission

In the only study to use MRI, Lukens et al. found good soft tissue definition, and were able to analyse ET opening with real-time turbo-gradient echo sequences during a Valsalva manoeuvre (Fig. 6) [24]. Although all patients had ETD, in unilateral cases, unaffected ears all appeared to open normally, whereas those affected did not.

**Table:** 

**Summary**: **What are the radiological features of obstructive ETD**? • While CT and MRI may demonstrate bony and soft tissue changes in some patients with obstructive ETD, there is yet to be a clinically useful or diagnostic imaging test	

### What are the radiological features of patulous ETD?

The defining feature of a patulous Eustachian tube is patency of the lumen at rest, when it should be closed. This condition has been successfully imaged using CT. Seven published records were identified, most including only very small case series [43–47], and only one reporting the results from case and control cohorts [31] (Table 2).

**Table 2:** Features of patulous ETD on imaging

Paper	Imaging modality	Cohort: patients (ears)	Basis for Patulous ETD diagnosis	Key findings
Tolley 1990 [43]	CT (no details)	4 (8)	CT only	4 patients with visibly patulous tube on CT—only one had symptoms, 3 had microsomia
Yoshida 2003 [44]	CT (1. cone beam seated; 2. standard CT lying flat, MPR)	2 (4)	Not stated	ET lumen long axis, short axis, cross-sectional area and total volume can be calculated Cartilaginous ET lumen larger in seated position when compared to supine
Yoshida 2004 [31]	CT (MPR)	20 (31) Cases 25 (50) Controls	Observed TM movement	Soft tissues visualised (poor views of Ostmann fat pad) 13/31 patulous ETs open throughout, 18/31 mostly open 0/50 controls open
Kikuchi 2009 [45]	CT (cone beam, 3D reconstruction)	35	Observed TM movement	Able to 3D reconstruct the patent lumen in most patients with patulous ETs
Yoshioka 2013 [46]	Cine CT (320-row area detector CT, reclining chair)	2 (3)	Observed TM movement or TTAG	ET patent prior to sniffing manoeuvre ET closes from the narrowest point towards the nasopharyngeal end Soft tissues seen moving upwards during closure
Oonk 2014 [47]	CT (Supine, no other details)	2	Observed TM movement	ETs bilaterally widely open along the entire length

*TM* tympanic membrane

**Fig. 7:**
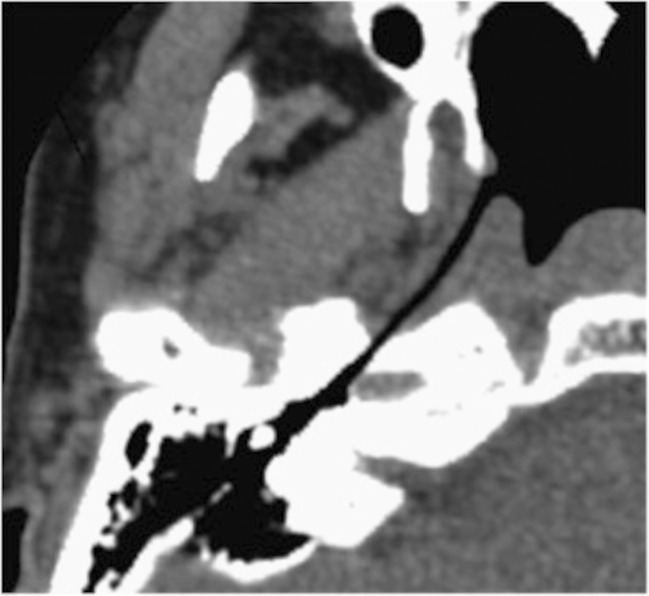
Oblique CT image of the ET and its surrounding tissues in a patient with severe symptoms of patulous ETD (reconstructed 1-mm thick, parallel and perpendicular to the long axis of the ET). The ET can be seen to be patent along its entire length. Taken from Yoshida et al. 2004 [31], with permission

The first published use of CT imaging was in 1990 by Tolley et al. who visualised tube patency in one patient with characteristic symptoms, and three patients with microsomia who were asymptomatic [43]. By reconstructing images in an oblique plane, several authors have been able to image the patent lumen over its entire length [44, 45, 47]. The most comprehensive study was published by Yoshida et al. in 2004, who examined whether patulous ETs were visible on CT scans. Of 31 patulous ETs examined, 13 appeared patent throughout their length, and the other 18 were seen to be mostly open. None of the 50 control (healthy) ETs examined were found to have visibly open lumens [31]. However, no mention is made of examiner blinding and the results have yet to be replicated. An image from the study is included (Fig. 7). The group also reported good definition of soft tissues, with the exception of Ostmann fat pad.

A drawback to the use of CT for imaging patulous Eustachian tubes is that patient symptoms often resolve when lying flat [4] suggesting that the patent tube closes. Because standard CT scanning is performed supine, there is a tendency to under-diagnose patulous ET using the technique. This problem has been overcome in two ways: either through the use of horizontal cone beam CT scanning with a seated patient [44, 45]; or with a tilting scanner and reclining chair, which facilitates positioning of the patient at 45 ° in a standard scanner [46]. Although the soft tissue contrast resolution of cone beam CT is inferior to standard CT, Yoshida et al. were still able to measure the Eustachian tube lumen long axis, short axis, cross-sectional area and total volume [44]. Interestingly, by using both cone beam and standard CT scanning techniques, this group was able to visualise closure of the abnormally patent lumen on supine positioning.

Rodrigues et al. took the CT scanning one step further in one patient, and used it to perform image-guided placement of a silicone elastomer suspension implant in the lateral wall of the ET, in order to narrow the ET lumen. Symptomatic improvement of patulous ET symptoms was reported [48].

**Table:** 

**Summary**: **What are the radiological features of patulous ETD**? • Abnormal patency of the ET lumen can frequently be seen on CT images using multiplanar reconstruction in a plane perpendicular to the long axis of the ET • Abnormal patency of the ET lumen can frequently be seen on CT images using multiplanar reconstruction in a plane perpendicular to the long axis of the ET	

### How can imaging be used as a measure of Eustachian tube function?

#### a) Clinical tests

The ultimate goal of imaging the ET has been to develop a technique that demonstrates whether the tube is competently performing its three main functions: middle ear ventilation, drainage of middle ear secretions, and prevention of sound and fluid reflux from the nasopharynx. Seven papers were identified describing the results from eight imaging methods that directly measured an aspect of ET function [49–55]. One paper was identified that described an indirect measure in the form of a correlation between lumen cross sectional area and tubal function [56]. Details of studies and results are summarised in Table 3.

**Table 3:** ET Function testing—obstructed ETD

Paper	Imaging modality	Route *Contrast*/ *isotope*	Cohort: patients (ears)	ET function tested *Patient action*	Non-imaging comparator		Key findings
*Direct*							
Parisier1970 [49]	Spot and cine x-ray	Intratympanic *Sodium diatrozoate*	10 perforations and -ve fluorescein clearance test	Patency/clearance *Swallowing*, *Valsalva*, *Toynbee*	Fluorescein clearance test	• Can visualise ET lumen and identify narrowings in the bony, junctional or cartilaginous portion	
			34 controls with TM perforations				
Bluestone 1971 [50] *part A*	Spot and cine x-ray	Intratympanic *Iophendylate*	24 Unrepaired CP 42 MEE 7 controls	Patency/clearance *VTs inserted*, *at rest*	None	• In repaired CP and unrepaired CP contrast failed to clear, or enter aural end of ET in most	
						• Contrast entry improved in all 2 weeks after VT insertion	
Bluestone 1971 [50] *part B*	Spot and cine x-ray	Nasopharynx *Sodium diatrozoate*	14 unrepaired CP 6 repaired CP 12 MEE and normal palate 5 healthy	Reflux protection *Swallowing*	None	• Contrast entered the ET and was ejected in healthy ears, but would not enter in unrepaired CP, and would often enter less far in repaired CP and MEE (blockage at the nasopharyngeal end)	
Gaafar 1988 [51]	Spot x-ray	Intratympanic *Lipiodol*	20 (32) Symptomatic ETD	Patency/clearance	Flexible endoscopy of nasopharyngeal orifice only	• The site of obstruction can be indicated in some, in others ET completely non-patent	
				*At rest and swallowing*			
Paludetti 1992 [52]	Scintigraphy	Intratympanic ^*77m*^ *TC*-*labelled albumin*	16 COM with TM perforations 2 controls	Patency/clearance *At rest*	Opening pressure manometry	• Slower ET passage of isotope in COM	
Brenner 1997 [53]	Scintigraphy	Nasopharynx and intratympanic ^*133*^ *Xe gas*	10 abnormal tympanogram 18 controls	Ventilation *Valsalva*	Tympanogram	• Lower median uptake and longer clearance half life in ETD	
Karasen 1999 [54]	Scintigraphy	Nasopharynx ^*133*^ *Xe gas*	16 ETD 13 controls	Ventilation *Valsalva*	Tympanogram	• Reduced isotope uptake in ears with ETD	
Celen 1999 [55]	Scintigraphy	Intratympanic ^*99m*^ *albumin*	32 MEE children with VTs 10 controls with dry perforations	Patency/clearance *At rest*	Tympanogram	• Passage of isotope in 16 % cases with MEE and 100 % controls • Longer to reach the ET and nasopharynx in cases	
*Indirect*							
Shim 2010 [56]	CT	None	80 COM 100 controls	Post-operative middle ear aeration	Otoscopy and tympanometry 1 year after surgery		• Cross-sectional area of the aerated bony ET may be useful for predicting the post-operative results
	(supine and prone)						

*CP* cleft palate, *TM* tympanic membrane, *COM* chronic otitis media (infection or inflammation of the middle ear), *VT* ventilation tube (grommet), *MEE* middle ear effusion

Three studies utilised spot and cine x-ray with a contrast agent injected into the middle ear via a perforation or grommet. It was demonstrated that the ET lumen could be visualised in healthy individuals, and that in patients with ETD, lumen narrowing could be seen either at localised points, or throughout the length of the tube [49–51]. Bluestone used the technique in patients with cleft palate and otitis media. He found that contrast failed to clear from the middle ear, or in some cases, even enter the opening of the ET within the middle ear, but that this improved significant only 2 weeks after grommet insertion [50, 57]. The same authors also used cine x-ray to examine the reflux of contrast introduced to the nasopharynx, and found that it entered the ET and was actively ejected in healthy individuals, but obstruction at the nasopharyngeal end prevented entry from the nasopharynx in patients with cleft palate or otitis media [50, 57].

Another imaging technique that has been successfully used to directly assess ET function is scintigraphy, with four studies published. Isotope-labelled human albumin introduced via a perforation in the tympanic membrane to the middle ear has been used to demonstrate reduced tube patency and clearance of middle ear secretions in ETD, with slower and incomplete passage of tracer along the tube [52, 55]. Labelled Xenon gas has been used to study middle ear ventilation by introducing it at both the nasopharyngeal and tympanic ends of the ET. It was demonstrated that less gas reached the middle ear during Valsalva in ETD patients, and that gas exchange back down the tube was also reduced [23, 24].

The final imaging technique that has shown promise in ETD case-control trials is an indirect measure of ET function. Shim et al. used CT to examine the ET of patients undergoing middle ear surgery [56]. They calculated the maximum cross sectional area of the aerated lumen in patients with chronic otitis media and not only found that it was significantly smaller than in healthy controls, but also that pre-operative size correlated with post operative success in the form of an aerated middle ear. They hypothesised that CT could therefore be used as a method of stratifying patients for surgical outcomes.

#### b) Research and developing techniques

The inaccessible nature of the ET means that imaging techniques have often been employed in research into its function. Some novel techniques remain potential future clinical tests, whereas others have simply been of use in determining the ET opening mechanism.

Early experiments in patients using nasopharyngeal or middle ear contrast, with multiple radiographs or cine x-ray, demonstrated reflux up the ET during opening when assisted by gravity, and suggested a pump-like action of the cartilaginous ET with closure from the tympanic end, clearing any contents into the nasopharynx in partnership with mucociliary clearance [58, 59]. Two groups have also attempted insufflating the ET with a mixture of air and powdered radio-opaque material, to provide evidence of ET patency and size, under non-physiological conditions [60, 61]. These techniques have not been developed further due to their complexity and poor images with radiographs. More recently, transtympanic injection of silver nanoparticles into the middle ear in an animal model has been shown to demonstrate middle ear and ET anatomy, and mucociliary clearance on CT images [62].

**Fig. 8:**
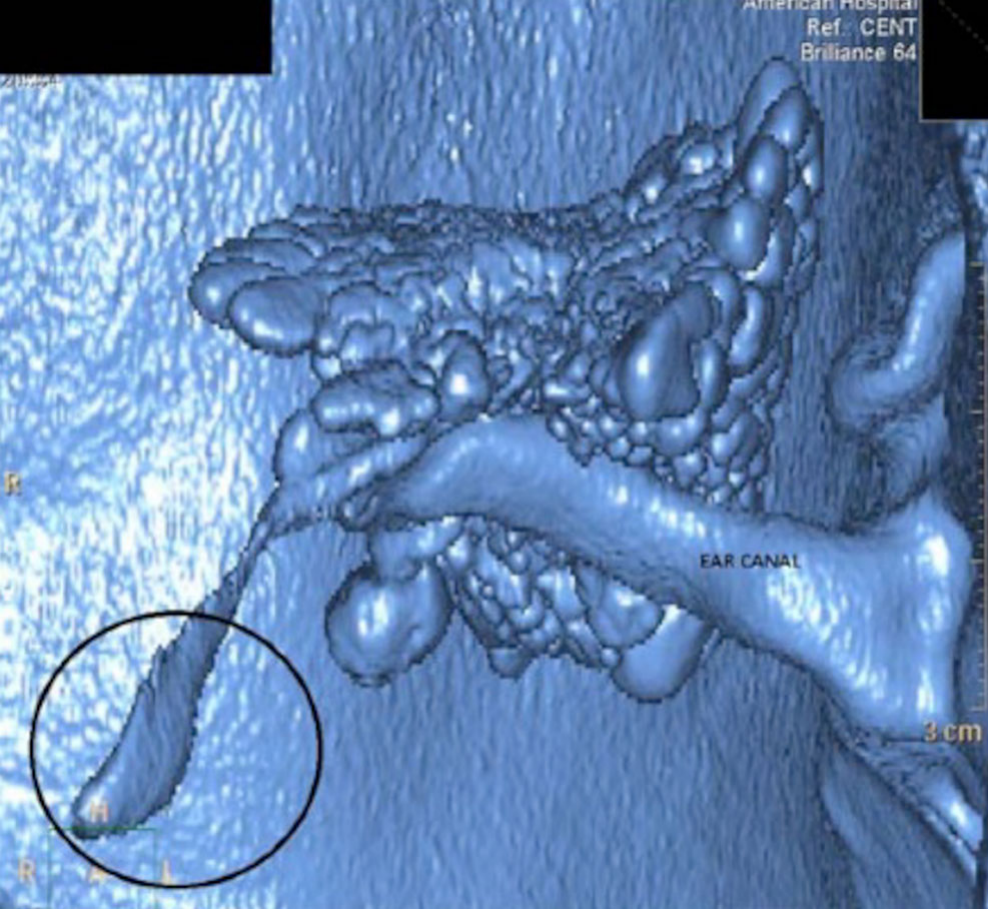
Three-dimensional reconstruction of the airspaces within the temporal bone from CT. Note the location of the isthmus of the ET and the relatively large distal aperture (*circled*). Taken from Tartabichi et al. 2015 [64], with permission

CT imaging has been utilised in the analysis of ET opening, which is still not fully understood. In healthy volunteers, Tarabichi et al. found that by performing CT during a Valsalva manoeuvre, the nasopharyngeal third of the cartilaginous ET could be seen in 94 %, and the whole length of the tube in 35 % [63]. Interestingly, the group went on to use the technique in ears undergoing surgery for chronic disease, and visualised a similar rate of patency in the nasopharyngeal third of the ET, suggesting that the obstruction may lie in the protympanic part [64]. A 3D CT reconstruction from the group is shown in Fig. 8. Another study comes from McDonald et al. who used cine CT to visualise an air bolus moving up the ET towards the middle ear [65]. While they were unable to follow the progress of a single opening over the entire ET length, the team hypothesised that their finding may explain why clinical tests of ET function suggest that the ET may not always open along its full length. This study examining gas exchange along the ET provides a clear contrast to other studies, which have looked at the clearance of secretions. Earlier work using radiographs suggested ET opening with progressive closure towards the nasopharynx as a method of pumping secretions out of the middle ear, and these findings were replicated by Niwa et al. who used intratympanic contrast with sequential CT repeats at 1 scan per second [66]. Interestingly, this group also tested the same ears with tubo-tympano-aerodynamic-graphy (TTAG), a manometric clinical test of ET opening during Valsalva. In a mixed patient group with chronic middle ear disease, they found that clearance and ventilation function did not always correlate with each other, suggesting that a single radiological or clinical test is unable to assess the function of the ET. As most middle ear pathologies arise from inadequate gas exchange across the ET, ventilation rather than clearance should be the focus of future imaging studies.

Karhuketo et al. successfully performed CT virtual endoscopy of the middle ear and osseous ET, though the closed passive state of the cartilaginous ET limits the usefulness of this technique outside the middle ear [67].

MRI has also been used to image the ET lumen opening. Krombach et al. asked healthy volunteers to perform Valsalva during image acquisition with a dynamic turbo-gradient echo sequence, and found that ET opening could be confirmed in 20 out of 22 ears [68]. The next step with this investigation would be to see if patients with ETD produce different results, and given the resolution of MRI, if areas or causes of lumen narrowing could be identified.

Patulous ETD may develop after weight loss or radiotherapy, but no anatomical cause for this has been identified. Two groups looked at Ostmann fat pad [27] and the ET cartilage shape [25] as potential candidates, but could find no relationship between MRI appearances and patulous disease. MRI has also been used to investigate the unknown mechanism of improved patulous ETD symptoms on lying supine. Oshima et al. measured the dimensions of the soft tissues around the ET on T1 contrast MRI before and during neck compression, and found that engorgement of the pterygoid venous plexus between the tensor veli palatini and the medial pterygoid muscles caused compression of the patent ET lumen from the antero-lateral side of the tube [69].

Dynamic anatomical imaging offers possible future applications. Yamawaki et al. used fast spin echo sequences to obtain T2 images to study dynamic changes induced by phonation [70]. They found that pharyngeal wall movements and ET cartilage movements seen with MRI correlated well with endoscopy findings, and hypothesised that rapid MRI may be a useful tool for the assessment of velopharyngeal function.

Given the ongoing uncertainty regarding the role of paratubal structures in ET opening and dysfunction, Leuwer et al. presented an interesting research technique using MRI voxel data to generate a 3D model of the ET and investigate muscle action [71]. Such modelling has been attempted before using histological sections [72–74], but the ability to perform this in vivo gives radiologists and otolaryngologists new opportunities for research into ETD.

**Table:** 

**Summary**: **How can imaging be used as a measure of Eustachian tube function**? (**a**) **Clinical tests** • Plain film radiograph and scintigraphy tests have shown the greatest promise, but have not been developed and therefore have not entered clinical use (**b**) **Research and developing techniques** • Imaging has greatly added to our understanding of ET function and research is ongoing • Dynamic imaging using new CT and MRI rapid acquisition techniques is beginning to provide new insight into ET function, and may yet provide a clinical test that allows diagnosis of ETD, and targeting of treatments	

## Discussion

The difficulties associated with clinically testing ET function were recognised over 50 years ago, when imaging was proposed as a non-invasive method of assessing function under more physiological conditions than could be achieved by using pressure based tests or catheterisation. The location, shape and orientation of the ET, and its mixed tissue composition, have meant that no imaging modality has proven to be superior overall.

The ultimate test of a new imaging technique is to assess its impact on the management of patients. Whilst one description of image-guided treatment was found [48], no identified publications described the use of imaging in clinical decision making for the identification and management of ET disorders.

The radiographic and scintigraphy tests that directly measure ET function could have potential for clinical use if developed, but despite many being described decades ago, they have not entered standard practice. This is in large part due to suffering the same problems that clinic-based tests of ET function face, such as a reliance on skilled and experienced technicians and in many cases, a requirement for a tympanic membrane perforation or grommet. If considered as a test for ETD, the sensitivity and specificity of identified imaging strategies would be poor in most cases, due in part to the heterogenous mix of pathologies and presentations linked to the condition [5].

The future of ET assessment, and in particular, functional assessment, probably lies in the development of CT and MRI techniques that can demonstrate both detailed anatomy and tubal opening, possibly with dynamic imaging using cine techniques becoming more routine practice. Even before new developments, it has been demonstrated that simple adjuncts such as asking patients to perform a Valsalva manoeuvre can provide some additional information.

In terms of research into ET function, we are reaching the limits of what can be gained from studying the static and often degraded tissues available from cadavers. It has been demonstrated, however, that there are still significant and clinically relevant gains to be achieved from the use of imaging to either directly visualise ET opening or to generate 3D models. In particular, these advanced imaging and modelling techniques have not yet been exploited in ETD patients and this may generate new knowledge that can lead to refined or novel interventions. Significant gains in understanding may also be achieved by comparing findings from imaging with clinical tests such as sonotubometry or tubomanometry, either performed in close succession or actually during image acquisition.

### Quality of existing studies

Even when case-control data were presented in identified studies, groups were small and often poorly defined. Although efforts at blinding were made by a few authors, most studies have a significant risk of bias and no controlled trials of any ET imaging techniques were identified. Where studies employed a case-control distinction, patient labels were applied prior to testing, and no published results have yet demonstrated the sensitivity or specificity of an imaging technique at assigning a diagnosis of ‘normal’ or ‘abnormal’ ET function to mixed population.

### Current hurdles and future developments

A problem shared with any ETD research or outcome measure development is the poorly defined nature of the disorder, which is due to the spectrum of possible presentations, and lack of a ‘gold standard’ test [75]. There will still be issues with generalisation, as clinical tests have shown different results in different populations [5], and particularly, in children ETD appears to differ in aetiology [2]. Current imaging work has focused on adults, but ETD is far more common in children. Imaging in the paediatric population will present new challenges when considering permissible radiation exposure and patient cooperation.

Patient positioning is an important consideration, as many newer imaging techniques require supine positioning, and as identified by some authors, this will affect ET function. Using a manometric method, it has been noted that even in healthy individuals, the volume of air passing through the ET is reduced by one third when the body is positioned at 20 ° to the horizontal and by two thirds when the body is supine [76]. Although cone beam CT perhaps provides the current best combination of spatial resolution and position, it is an area for development.

Imaging and reconstruction protocols to optimise orientation, tissue definiton and capture ET movement need to be futher developed. Through collaboration between radiologist and otolaryngologists, it is possible that the use of intranasal or intratympanic contrast may again prove of benefit with the progression of cine CT and MRI techniques.

Most studies identified to date have made qualitative assessments of opening, although anatomical measures of size and volume have been possible. For maximum clinical utility, quantifying ET opening or function is the goal. Clinical tests are beginning to reach this point, though no single investigation has declared itself as superior; and currently, diagnoses are mostly made on expert opinion. For the foreseeable future, it is likely that the assessment of patients with obstructive or patulous ETD will reply on a combination of imaging, clinical tests, patient reported measures and expert opinion.

## Conclusions

ET imaging is increasingly important given the renewed interest in ET disorders generated by novel clinical tests and emerging surgical treatments. No single imaging modality has proven sufficient when examining ET anatomy or function; but if chosen carefully, certain protocols can offer radiologists and otolaryngologists significant useful information. A review of the literature has demonstrated that currently, there is insufficient evidence that imaging should be part of routine ET assessment, and none of the techniques designed to assess ET function can be recommended for use outside of a research setting. However, MRI and CT can provide significant anatomical detail. New high-resolution and rapid-acquisition MRI and CT scanning techniques are under development, and may still provide a clinically useful, non-invasive test of ET function.
